# *Plasmodium falciparum* malaria and invasive bacterial co-infection in young African children: the dysfunctional spleen hypothesis

**DOI:** 10.1186/1475-2875-13-335

**Published:** 2014-08-26

**Authors:** Gloria P Gómez-Pérez, Robin van Bruggen, Martin P Grobusch, Carlota Dobaño

**Affiliations:** Barcelona Centre for International Health Research (CRESIB, Hospital Clínic-Universitat de Barcelona), Barcelona, 08036 Spain; Centre of Tropical Medicine and Travel Medicine, Department of Infectious Diseases, Academic Medical Centre, University of Amsterdam, Meibergdreef 9, PO Box 22660, 1100 DD Amsterdam, The Netherlands; Department of Blood Cell Research, Sanquin Research and Landsteiner Laboratory, Academic Medical Center, University of Amsterdam, Plesmanlaan 125, 1066CX Amsterdam, The Netherlands

**Keywords:** *Plasmodium*, Malaria, Invasive bacterial infection, Children, Marginal zone B cells, Hyposplenism, Spleen

## Abstract

Children with recent or acute malaria episodes are at increased risk of invasive bacterial infections (IBI). However, the exact nature of the malaria-IBI association is still unclear. Young children have an age-related spleen immunologic immaturity, mainly due to the still ongoing development of the marginal zone (MZ) B cell subset. By mounting a rapid antibody response against encapsulated bacteria, these cells are critical for the defence against highly pathogenic microorganisms that do not elicit classical T cell-dependent responses. There is increasing evidence that the anatomy of the spleen becomes disorganized during malaria infection, with complete dissolution of the MZ and apoptosis of MZ B cells. Correspondingly, a reduction in the frequency of the peripheral equivalent of the MZ B cells has been found in malaria endemic areas. A remarkable similarity exists in IBI susceptibility between African children with malaria and hyposplenic or splenectomized patients. However, studies specifically assessing the immune function of the spleen in controlling bacterial infections in young children with malaria are scarce.

Here, it is hypothesized that *Plasmodium falciparum* malaria infection constitutes a detrimental factor in the still immature spleen function of young children, resulting in a factually hyposplenic state during malaria episodes, putting children with malaria at a high risk to develop life-threatening bacterial infections. Studies to confirm or reject this hypothesis are greatly needed, as well as the development of affordable and feasible tools to assess the immune spleen function against encapsulated bacteria in children with malaria.

## Background

For years, in malaria-endemic settings the percentage of children with splenomegaly has been correlated with intensity of malaria transmission in a community. The method to evaluate malaria endemicity by spleen measurement is named the Hackett score, and it was introduced by Dempster in India in 1848 before it was known that *Plasmodium* species were the causative agent of malaria. Later, this method became accepted by the World Health Organization to be used in malaria surveys [1, 2].

**Figure 1:**
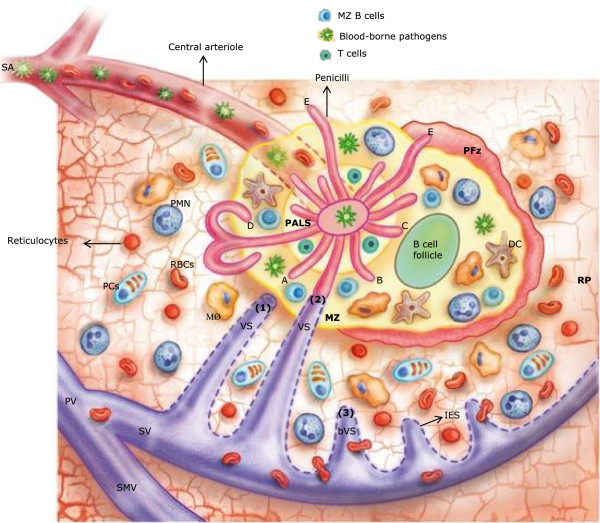
**Structure of the human spleen.** Adapted from Bowdler [6]. Arterial blood enters the spleen through the splenic artery (SA) that branches in multiple arterioles. Central arterioles are surrounded by periarteriolar lymphoid sheaths (PALS) that contain the T cell zone. Next to the PALS are the B cell follicles. Together, PALS and B cell follicles constitute the white pulp of the spleen (WP). Most of the arterial blood that enters the spleen is drained into the marginal zone (MZ) through small open ended capillaries named penicilli. Ninety per cent of the blood that enter the MZ is drained directly into (1) open ended venous sinuses (VS) or (2) penicilli-VS connections, constituting the fast pathway. Ten per cent of the blood is drain into the open circulation of the perifollicular zone (PFz) and red pulp (RP) constituting the slow pathway. Normal red blood cells (RBC) need to pass into (3) blind-ended VS (bVS) through interendothelial slits (IES) to go back to the peripheral circulation, VS drain into the splenic vein (SV), and the latter into the portal vein (PV) after joining the superior mesenteric vein (SMV). PCs: plasma cells; PMN: polymorphonuclear neutrophils.

The spleen is a complex lymphoid organ with several important functions that starts its development in foetal life and reaches full maturation during early childhood, around age two to three years [3–5]. The characteristic that makes this organ unique is that it is the only lymphoid organ specialized in the filtration of blood, while the rest of lymphoid organs filter lymph. Additionally, the spleen contains the largest single aggregate of lymphoid tissue in the body, housing approximately one third of the total circulating lymphocytes, thus with a vast number of them migrating through the spleen at any given time, surpassing the combined traffic of all lymph nodes in the body [6]. It is also in the spleen where a large population of naïve B cells produced in the bone marrow matures into memory B cells. Overall, a special attribute of the spleen immune function is its capacity to mount T cell-independent (TI) immune responses against polysaccharide and lipopolysaccharide micro-organism antigens in non-immune individuals (TI-1 and TI-2 responses, respectively). This response can take place within 24 to 72 hours after encountering bacteria or other pathogens by phagocytosis and readily production of IgM [6–8]. Hence, the spleen function ‘fills in’ the time gap between the innate and the adaptive immune response, with the latter taking several days to develop. The filtration of bacteria and their destruction in the spleen must be a rapid process in order to overcome the speed of replication of these micro-organisms [6], a process for which the spleen is fully equipped and in which absence, life-threatening invasive bacterial infections (IBI) can enfold. Therefore, the spleen represents the second line of protection against microbes when they manage to breach the first line of protection, the mucosal barrier [9]. The unique structure of the microvascular pathways of the spleen (Figure 1) reflects its two most important functions: (1) the removal of senescent and damaged red blood cells (RBCs); and, (2) the removal of blood-borne micro-organisms and cellular debris [10–12]. Both functions involve an intense phagocytic activity that occurs in different compartments of this organ by different sets of immune cells.

To accomplish its functions, the spleen encompasses the following anatomic subunits (Figure 1): (1) the white pulp (WP), containing T cell zones (periarteriolar lymphoid sheath (PALS)) and B cell follicles, where the adaptive immune response takes place; (2) the marginal zone (MZ) containing macrophages, dendritic cells (DCs), natural killer T (NKT) cells (in mice), B cell-helper neutrophils (N_BH_), innate lymphoid cells type 3 (ILC3), CD4^+^ T lymphocytes, MZ B cells and memory B cells, where TI-1 and TI-2 responses take place; (3) the perifollicular zone (PFz), separating the MZ from the red pulp (RP) and containing RBCs, pericapillary macrophages, N_BH,_ and ILC3; (4) the RP-containing macrophages, DCs, N_BH_, plasmablasts, being the place where RBCs are efficiently filtered from the circulation and where reticulocytes mature [9, 10, 13–16]. Five to six per cent of the cardiac output of 5–6 L per minute in adults flows through the spleen [17, 18]. Most of the blood flow is deposited in the MZ through capillaries branched from splenic central arterioles (Figure 1). The small capillaries in the MZ called penicilli distribute part of the blood flow to the follicles, the PFz and the RP [6]. Penicilli have different possible ends (Figure 1): (A) and (B) the MZ at different points; (C) penicilli can enter the MZ and curved to end into the B cell follicle; (D) they could reach the RP and curve back to the external border of the MZ; (E) or they could drain directly into the PFz and the RP. Blood components, possibly containing foreign antigens, are sorted in the MZ by cell-cell interactions with the immune and endothelial cells located in this zone, to the WP or to the RP. Distribution of the blood cells is not random. Ninety per cent of the blood flow coming out from the MZ is drained directly into open ended venous sinuses (VS) or penicillin-VS connections, and from there to the splenic venous circulation constituting the fast pathway. Ten percent of the blood is drained into an open circulation system in the PFz and the RP (slow pathway) [6, 18]. The RP is constituted by a reticular meshwork with pulp cords that form a three-dimensional web where specialized macrophages reside. Here, in the slow pathway, immature RBCs (reticulocytes) and abnormal RBCs adhere to the meshwork mediated by adhesion molecules, or are being captured by macrophages due to specific signals of immaturity, aging or damage, and no longer circulate. Blood-borne pathogens and debris can also be captured in the RP. Normal RBCs pass through the RP towards the inter-endothelial slits (IES) on the walls of the blind-ended VS located in this area, trespassing into the VS lumen and going back into the peripheral circulation (Figure 1). All this ‘stickiness’ slows down the speed of RBC flow into the VS, because even normal RBCs transiently adhere to the reticular meshwork, reaching the RP haematocrit concentrations as high as two-fold the arterial haematocrit (~80%), representing 75% of the spleen volume in normal conditions [6]. Damaged senescent RBC can also be captured in the RP by mechanical retention. Nevertheless, the 90 to 10% differential distribution of the blood flow in the spleen to the lymphocyte-RBC compartments, respectively, suggest that the immunological function of the spleen takes priority over the filtration of blood cellular elements in the RP [6]. Importantly, all this extremely organized biophysical distribution of the blood and its cellular components into the different subunits of the spleen is disrupted in malaria infection [17, 19, 20].

*Plasmodium falciparum* malaria causes alterations in the function of the cells of the innate immune system such as macrophages [21], neutrophils [22] and dendritic cells [23]. It also produces changes in the adaptive immune system, altering B cell populations [24–26] and the response to certain vaccines [27]. Regulatory cytokines released during acute infection aiming to control the pro-inflammatory response against malaria parasites [28] could impair the mucosal immune response to invasive non-typhoid salmonellae (NTS) infections [29]. Currently, there is increasing evidence in the scientific literature that young children (<three years old) infected with malaria might suffer from functional hyposplenism. Here, it is hypothesized that *P. falciparum* infection impairs the immunological response of the spleen against encapsulated bacteria in young children, a postulate built upon the following observations.

### Observation 1. Children with malaria are similar to splenectomized patients or patients with hyposplenism regarding their susceptibility to IBI

One of the risk factors to develop IBI in Africa is *P. falciparum* malaria [30–33]. Bacteria responsible for these invasive infections in patients with malaria are NTS, *Streptococcus pneumoniae, Haemophilus influenzae* type b, *Staphylococcus aureus, Escherichia coli* and other gram-negative bacteria [30–32, 34–37]. Reported specific risk factors to develop NTS bacteraemia are: younger age, acute and recent malaria infection, severe malarial anaemia (SMA), splenomegaly, human immunodeficiency virus (HIV) and severe malnutrition [32, 33, 36, 38–44]. Therefore, it is well established that African infants younger than 36 months bear the brunt of malaria infection, presenting with the highest rates of IBI co-infection compared with older children and HIV-negative adults [33, 42, 43]. Remarkably, splenectomized patients or those with hyposplenic states are well known to be susceptible to develop serious IBI by this same group of bacteria [45–55]. For example, it is common knowledge that splenectomized patients are susceptible to overwhelming post-splenectomy infection syndrome (OPSI), which consists of a fulminant sepsis mainly caused by *S. pneumoniae*, characterized by a particularly high mortality [45]. Of note, among splenectomized patients, infants and young children are at higher risk for IBI than adults [54]. The reason is that young asplenic children have low pre-existing circulating antibodies and, in the absence of the spleen, are unable to mount a sufficiently fast antibody response against intravascular antigens and to phagocytose antibody un-opsonized bacteria while the adaptive immune response is under development [6]. Conversely, the splenectomized adult population tends to have pre-existing antibodies due to a larger immunological memory repertoire compared to very young children, overcoming the necessity for developing a fast immune response and controlling the infection. These immune complexes in the absence of the spleen can then be phagocytosed in the liver by Kupffer cells [6, 56], although less efficiently than in the spleen, explaining the occurrence of IBI also in adult splenectomized patients. A very well studied hyposplenic population is sickle cell disease (SCD) individuals [11, 45, 49, 55, 57–59]. Interestingly, as in the case of splenectomized children, infants and young children with SCD are at higher risk of IBI when compared to the adult population [57]. Children with SCD (homozygous genotype) under five years of age present IBI by *S. pneumoniae* at rates 30–100 times higher than those expected in healthy children from the same age group [58]. Splenic dysfunction is the foremost contributor to the enlarged risk.

### What are the underlying immunological mechanisms that make hyposplenic subjects, especially young children, susceptible to IBI?

#### Opsonization is impaired in the absence of spleen

Routinely, hyposplenism has been diagnosed by haematological rather than immunological methods [6]. Nevertheless, Lammers et al. [11] have performed a study in SCD patients (homozygous (SS) and heterozygous (SC) genotype) and splenectomized patients, aiming at correlating the results of spleen haematological function tests with conventional methods, to the immunological spleen function and peripheral B cell subsets in these different hyposplenic individuals as compared to control subjects. The methods used to assess the haematological function of the spleen were (i) Howell Jolly bodies (HJB) assessment; (ii) pitted red cell count; and, (iii) ^99m^Tc-labelled autologous RBC scintigraphy to measure functional splenic volumes (FSV). The immunological function was assessed by studying the response to polysaccharide vaccines. As expected, SS and SC individuals had a significantly diminished anti-polysaccharide response. Interestingly, HJB were indicative of splenic dysfunction; however, its absence did not necessarily indicate a normal functioning spleen tissue. Even more interesting, the FSV was strongly correlated with the amount of MZ B cells in peripheral circulation, being significantly reduced in SS and splenectomized patients, thus corroborating previous findings [60]. These results indicate that the MZ B cell subset population might be reduced in the spleen of hyposplenic individuals, with the consequently impaired immune response to encapsulated bacteria and reduced IgM response. These results are in correlation with the well-established functional opsonization failure in hyposplenic patients [58, 61, 62]. An illustration of this defective opsonization is found in a clinical case report of a patient with SCD who presented with refractory septic shock. Despite aggressive therapy, the patient remained on two vasopressors and yielded persistent bacteraemia. Within one day of starting intravenous g-globulin, vasopressors could be discontinued, indicating a quick response to the g-globulin infusion as a rescue therapy [63].

Additionally, in the spleen tuftsin is synthetized, an opsonin that activates the alternative complement pathway [6, 17], and that has receptors expressed on monocytes/macrophages, neutrophils and NK cells [64]. Upon binding its receptor, the complex is taken into the phagocyte and initiates the production of superoxide and nitroxide radicals, essential molecules for the killing of phagocytosed bacteria [64]. Therefore, the main outcome of tuftsin binding to macrophages and neutrophils is the stimulation of the ability of these cells to digest that leads to termination of phagocytosis in lymphoid formations [64]. Its importance has been confirmed by the development of fatal infections in splenectomized humans and other animals with lack of tuftsin [64]. Moreover, serum tuftsin concentrations are decreased in SCD subjects, causing a functional defect in the alternative complement pathway [65, 66]. Importantly, tuftsin derivatives and analogues such as polytuftsin and rigin have been tested as therapeutic tools in several macrophage-based infections [67], showing to increase anti-tuberculosis drug treatment uptake by macrophages [68]. Tuftsin has also been used as a malaria and influenza A vaccine adjuvant [69–71], as a prophylactic in malaria animal models improving infection control [72], and as an immune-stimulating molecule along with antifungal agents in the treatment of opportunistic fungal infections [73]. In addition, combined anti-leishmanial drug treatment with tuftsin increases *Leishmania* parasite phagocytosis by macrophages [74], and when used as adjuvant treatment it helps to control sepsis in a murine model [75].

#### Neutrophil and monocyte dysfunction

Patients with malaria infection and SCD present with neutrophil dysfunction, characterized by defective locomotion, phagocytic processes and bactericidal performance [22, 66, 76], related to a poor or almost absent oxidative activity in a subpopulation of neutrophils [66]. Additional studies show similar alterations in the monocyte subsets as well, related to haemolysis itself, but overall due to a direct toxic effect of iron liberated from malarial pigment (haemozoin) and through adhesion of infected RBC (iRBC) to monocytes, macrophages and myeloid DC surfaces [21, 23, 76, 77]. Therefore, patients with SCD and malaria, as well as patients with other haemolytic diseases such as bartonellosis, are unusually susceptible to bloodstream invasion with *Salmonella* spp. [78]. In children with mostly (95%) uncomplicated *P. falciparum* malaria, with ages between four and eight years, it was found that the neutrophil dysfunction was a consequence of induction of a haem-degrading enzyme (haem oxygenase-1) in neutrophil progenitors in the bone marrow [22]. The dysfunction in oxidative burst activity did not affect the whole neutrophil population and it was long-lasting, recovering normal function only eight weeks on average after a malaria episode. However, in this study it was not possible to establish the threshold of proportion of dysfunctional neutrophil population needed in blood in order to increase the susceptibility of malaria patients to NTS bacteraemia. Neither was it possible to explain why young children are more susceptible to NTS systemic infections than immune-competent adults, since neutrophil dysfunction due to falciparum malaria could also occur in the adult population during malaria episodes. As the phagocytosis by mononuclear cells and neutrophils is enhanced in the presence of antibodies, and in SCD patients bacterial phagocytosis is substantially improved with immunoglobulin (Ig) treatment [63], here it is proposed that neutrophil dysfunction may be contributing to the increased susceptibility to IBI in young children with malaria [22], but it is not the most important factor.

### Observation 2. MZ B cells are decreased in children with malaria

As aforementioned, one of the anatomic subunits of the spleen tissue is the MZ. The MZ of the spleen is strategically positioned between the lymphoid compartment of the spleen, the WP, and the more innate, scavenging RP compartment (Figure 1). Most of the arterial blood that enters the spleen runs through the MZ inhabited by a special set of cells, such as macrophages expressing unique combinations of pattern recognition receptors, as well as MZ B cells that can be readily activated [19]. MZ B cells are a distinct B cell lineage [79] with a unique surface phenotype, expressing polyreactive IgM (B cell receptor: BCR), IgD, CD1c, CD35 (complement receptor 1 (CR1)), CD21 (complement receptor 2 (CR2)), CD27, high levels of Toll-like receptors (TLR), MHC class II, and TACI (an Ig-inducing receptor) [13, 80]. Through the dual binding of antigens to the BCR and TLR, MZ B cells are promptly activated, undergoing Ig class switching and somatic hyper-mutation in a T cell-independent manner. In mice it has been shown that under certain stimuli, and using CR1, CR2 and the chemotactic receptors S1P1 and CXCR5, MZ B cells migrate, shuttling antigens to the B cell follicles [81], returning afterwards back to the MZ. When only the BCR is stimulated, MZ B cells migrate to the T cell zone to develop a T cell-dependent antibody response. Through TACI receptors, MZ B cells receive assistance by the N_BH_ to produce IgM and for class switching from IgM to IgG or IgA in a T cell-independent manner [16]. Other important populations in the MZ are the already mentioned macrophages as well as DCs, ILC3 and switched memory B cells [9]. NKT cells have been found in human spleen tissue [82]; however, its localization within the spleen is not clear. In mice on the other hand, NKT cells have been found in the MZ and RP initiating protective immune responses against blood-borne antigens [14].

Depletion of cells in the MZ during acute malaria infection was reported in the *Plasmodium chabaudi* AS infection mouse model [83]. Later on, Urban and collaborators found a marked dissolution of MZ with loss of B cells in human spleens of adult patients dying from *P. falciparum* malaria although not significant [84]. Recently, it was shown that acute *P. chabaudi* AS infection in mice causes apoptosis of transitional T2 and MZ B cells during and subsequent to the control of the first wave of parasitaemia [85]. These findings have been corroborated by several reports of apoptosis of different immune cells in the spleen in malaria animal models [86–89]. A proposed underlying mechanism of reduced B cells in the spleen is the impaired generation of bone marrow MZ B cell precursors. Regarding the malaria-driven apoptosis mechanism, it has been proposed to be mediated by pro-inflammatory cytokines such as TNF [13], functional exhaustion of the MZ due to abnormally high influx of parasitic antigens [12, 13], and the synergy between the accumulation of free haem in plasma during *Plasmodium* infection and TNF production, that mediates programmed cell death in immune cells [90].

As explained, the development of peripheral B cells takes place during infancy. However, in malaria-endemic settings some alterations in B cell subsets independent of age have been reported [91]. For instance, a study performed in western Kenya found suppression of circulating IgD^+^CD27^+^IgM^+^ B cells (the peripheral equivalent of MZ B cells) in infants aged one to 24 months living in a malaria-endemic region when compared with a similar infant population from an unstable malaria transmission region [91]. Supporting these findings, it has been reported that four- to five-year old children in Uganda with the highest incidence of malaria in the prior year of the study (> = seven episodes/year, n = 20) exhibited a transient drop in frequencies of IgD^+^CD27^+^IgM^+^B cells at the time of acute malaria, not seen in subjects with a lower incidence [92]. A recent study performed at the Barcelona Centre for International Health Research (CRESIB) also shows a decrease of MZ-like peripheral B cells in pregnant women from Papua New Guinea with life-long exposure to *Plasmodium* infections (Requena et al.*,* in press *J Immunol*). Here, it is hypothesized that the drop of the MZ B cells in peripheral circulation of patients with malaria is a result of the disruption in spleen architecture observed in the referred histology studies. Furthermore, the MZ B cell deficiency along with some other possible defective immune spleen functions appears to be responsible for a transient hyposplenism in children with malaria, increasing their risk to IBI. Importantly, children with malaria have been reported to show a lower antibody response against group C meningococcal polysaccharide vaccines when compared to children from the same setting but without parasitaemia, supporting this hypothesis [27]. In line with this finding, immunological memory against previous vaccinations, including diphtheria toxoids, is lost in animal models after malaria episodes by deletion of memory B cells and long-lived plasma cells in spleen and bone marrow [93].

Taking into account that the spleen MZ has not yet reached maturity in young children, it can be speculated that the threshold of MZ dysfunction (due to falciparum malaria or other pathologies) that is needed to develop immune hyposplenism is lower in children when compared to the adult population, probably explaining children’s increased susceptibility to bacteraemia in the context of malaria infection when compared to the immune-competent adult population.

It is important to clarify that the peripheral equivalent of the MZ B cells have different nomenclatures in the research literature: MZ-like B cells, IgM memory B cells [94], splenic MZ B cells (S-MZ B cells) [7], IgD + IgM + CD27+ B cells, IgD + CD27+ B cells [91] and CD27 + IgM + B cells.

### Observation 3. Antibody and complement are necessary to control NTS invasive infection

NTS bacteraemia most commonly occurs in African children below two years of age. A relative sparing of infants younger than four months old has been found coincidently within the period during which maternal antibodies are still present [95]. Sera from healthy children less than 16 months old were shown to lack NTS-specific antibodies, and sera lacking antibodies did not kill NTS despite normal complement function [96]. Additionally, *Salmonella*-specific antibodies facilitate specific T cell responses via augmentation of bacterial uptake and induction of apoptosis in macrophages [97]. Moreover, transferred low- and high-affinity IgG2c, and with less efficiency IgM, were shown to impede *Salmonella* colonization of splenic macrophages, markedly reducing bacteraemia by antibodies induced during the infection in mice [98]. Therefore, as the fast antibody response against encapsulated bacteria is developed in the spleen, here it is postulated that malaria-induced hyposplenism could be responsible for a defective immune response against NTS and other encapsulated bacteria in children with malaria, substantially increasing their risk to develop IBI.

### Observation 4. Children with SMA have a higher prevalence of IBI co-infections than children with cerebral malaria

A study in Sudanese children reported that SMA was associated with a larger spleen, longer fever duration and lower parasitaemia than cerebral malaria (CM) [99]. In these children, SMA and CM clinical presentation differed significantly in several aspects, including median age (three versus six years), prevalence of splenomegaly (57 versus 11%), and spleen size (Hackett grade 3.5 versus 0). Recently, a study reported that spleen volume measured by ultrasonography was lower in children with CM compared with children with SMA [100]. Interestingly, similar results were found some decades ago in an animal model of malaria infection in which resistant mouse strains developed marked splenomegaly and survived the infection, whilst susceptible mice succumbing to the infection developed only minimal splenomegaly [83]. In humans, SMA is more prevalent in children younger than two years old in holo-endemic areas [18, 99, 101]. Therefore, there is an age-dependent variation of iRBC clearance, with older patients having less efficient parasite clearance than young children, suggesting an innate retention process [99]. Previous findings reported that patients with malaria and splenomegaly markedly accelerated clearance of ^51^Cr-labelled RBC and presented with a lower mean haematocrit than patients without splenomegaly [102]. Filtration-based parasite clearance is likely the key to spleen-dependent innate protection [99]. However, how this innate function of the spleen protects against malaria parasites and how the retention of iRBC and uninfected RBC (uRBC) in this organ influences immunologic response in the young host has not yet been studied.

**Figure 2:**
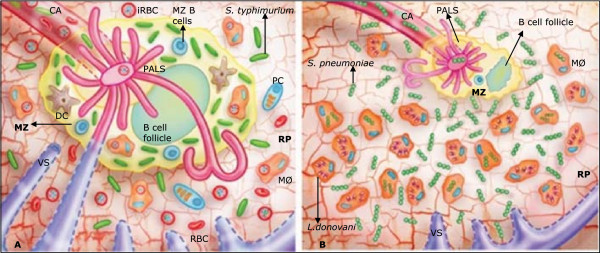
**Parasite and bacteria co-infection in the rodent spleen.** **A.** *Plasmodium chabaudi – Salmonella typhimurium* co-infection in the rodent spleen. Adapted from Yavada et al. [109]. The white pulp (periarteriolar lymphoid sheaths (PALS) and B cell follicle) and the marginal zone (MZ) do not show atrophy and preserve their function. **B.** *Leishmania donovani – Streptococcus pneumoniae* co-infection in the rodent spleen. Adapted from Kirby et al. [110]. The white pulp and the MZ are notoriously atrophic, showing great amounts of macrophages with phagocytosed amastigotes of *L. donovani*. In the absence of MZ, *S. pneumoniae* localized in the red pulp (RP) where RP macrophages compensate the MZ dysfunction. CA: central arteriole; VS: venous sinuses; RBC: red blood cell; iRBC: infected RBC; MØ: macrophages; PC: plasma cells; DC: dendritic cells; MZ B cells: marginal zone B cells. The perifollicular zone (PFz) was not drawn in these Figures.

**Figure 3:**
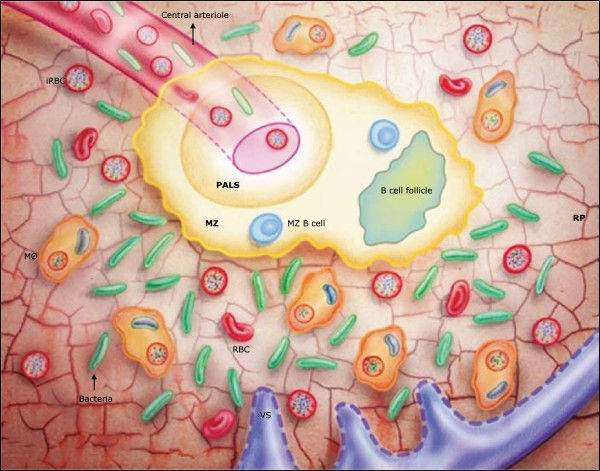
***Plasmodium falciparum*** **and invasive bacterial co-infection in the human spleen.** The marginal zone (MZ) during *P. falciparum* malaria is dissolved, therefore lacking its most important armamentarium against encapsulated bacteria. The red pulp (RP) macrophages phagocytic capacity is overload by erythrophagocytosis, and in the presence of infected erythrocytes, macrophages will deficiently phagocytose bacteria. Bacteria replicate rapidly causing bacteraemia. (MØ) macrophages; (VS) venous sinuses. Penicilli and perifollicular zone (PFz) were not incorporated into this Figure.

Of note, several studies have shown that children with SMA have a higher prevalence of IBI co-infections (largely NTS bacteraemia) compared with children with CM who infrequently have bacterial co-infections [32, 33, 103]. The increased malaria parasite clearance in SMA would explain the reported tendency of children with malaria and IBI co-infection to have lower parasite densities [31, 33, 103], as well as the low frequency of NTS infection in children with high malaria parasite counts [43, 104]. Therefore, the ‘SMA-IBI-young age’ association needs to be elucidated. A possible innate mechanism that could explain the prevalence of SMA in young children is the age-related change in RBC complement regulatory proteins (CRP) [101, 105, 106]. The detailed discussion of this possible mechanism is beyond the scope of this manuscript. Briefly, CRPs are in low concentration on the surface of RBC in young children, increasing the splenic erythrophagocytosis of iRBC and uRBC during malaria infection, as compared to the adult population [107, 108], with the consequent development of splenomegaly. It has been found that splenomegaly in malaria patients is the result of splenic accumulation of iRBCs, uRBCs, macrophages, and reticular cells [99], among other cells. It was mentioned earlier that under normal conditions the splenic RP reaches a haematocrit of 80%. Therefore, during malaria infection, the increased amount of entrapped RBCs in the RP requires some tissue rearrangements. Two animal models illustrate two different possible situations in the presence of *Salmonella typhimurium* or *S. pneumoniae* bacteria with parasitic co-infection of the spleen. Apparently, co-infection by a non-lethal *Plasmodium* strain and *S. typhimurium* in mice does not alter the compartmental distribution of malaria iRBC and bacteria in the spleen [109]. Malaria iRBC in these experiments were located in the RP, and bacteria were located in the MZ (Figure 2A). Taking all limitations of the mouse model into account, both micro-organisms were successfully cleared, demonstrating conserved haematological and immunological spleen functions despite the concomitant infections. Another research group decided to test the bactericidal function of the spleen in the absence of MZ. Mice with chronic *Leishmania donovani* infection (which presents with progressive loss of macrophages in the MZ in direct relation to splenomegaly) were co-infected with *S. pneumoniae*. In this case, in the absence of MZ macrophages, bacteria were located in the RP instead of the MZ, and phagocytosed by RP macrophages, showing a relocalization of the bactericidal functions in the spleen [110] (Figure 2B). However, here it is postulated that in the case of invasive bacterial co-infection with a lethal malaria parasite strain, such as *P. falciparum*, this relocalization will not occur due to overload of RBC in the RP, among other factors. In human *P. falciparum* malaria, similarly to the *L. donovani-S. pneumoniae* co-infection, the MZ would be significantly reduced. Nevertheless, some important differences with this animal model would be: 1) the chronicity of infection in this *L. donovani* animal model, in contrast with acute *P. falciparum* malaria in humans, perhaps implying that the total phagocytic capacity of the spleen RP is not necessarily overwhelmed in chronic *Leishmania* infections - even though in massive splenomegaly cases of *L. donovani* in humans, the important structural changes in the spleen might impact its immunological function; 2) the high parasitaemia and splenic overload with iRBC and uRBC in the case of *P. falciparum* malaria, added to the increased erythrophagocytic capacity in young children, that will demand maximum phagocytic capacity from the macrophages in the RP and the MZ [111] (Figure 3). Importantly, in the case of falciparum malaria, even if bacteria reach the RP, the chances to be phagocytosed there will be low. The reason is the evidence arising from experiments performed in in vivo animal models and in vitro [78]. These studies show that macrophages simultaneously exposed to damaged RBC and *S. typhimurium* organisms preferentially phagocytose damaged RBC, presenting a defective bactericidal capacity. The reasons are not clear. It is postulated that inhibition of bacterial phagocytosis may be mediated by competition mechanisms between damaged RBC and bacteria for phagocytic sites on macrophages, or that macrophages are just overloaded temporarily [111].

### Putting two and two together

**Figure 4:**
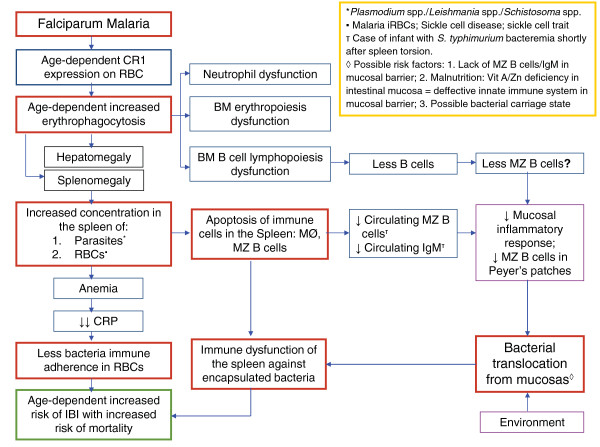
**Summary of possible key events leading to bacterial co-infection in young children with** ***Plasmodium falciparum*** **malaria.** RBC: red blood cell; BM: bone marrow; MZ B cells: marginal zone B cells; MØ: macrophages; IBI: invasive bacterial infection; CRP: complement regulatory proteins.

Synthesizing the above information, it is evident that due to innate mechanisms, African children younger than three years old develop SMA with accompanied splenomegaly more frequently than older children and adults. The changes in the immune cell architecture of the spleen under these circumstances might affect the immunological function of this organ against encapsulated bacteria. Initially by disruption and almost disappearance of the MZ and its important fast bactericidal function, but also by impairing other spleen functions like the production of tuftsin, presenting opsonization failure and defective function of the alternative complement pathway. In *P. falciparum* infections, the phagocytic capacity of the spleen in young children might be saturated, with RP macrophages preferentially phagocytosing iRBC over bacteria [78, 111]. As a consequence, in the case of a bloodstream invasion of bacteria at the same time as *P. falciparum* infection, bacteria will find the MZ almost inhabited and the RP macrophages busy and skewed to iRBC and uRBC phagocytosis. Subsequently, bacteraemia takes place (Figure 3 and 4).

## Additional mechanisms

### Bacterial carriage status

It is highly likely that children who develop malaria-IBI co-infection are carriers of the causative bacteria when they develop malaria infection. There is evidence that 6.9% of household contacts of children with IBI by NTS are asymptomatic carriers of NTS in the stools, including healthy children [112]. Additionally, NTS seems to behave like an opportunistic infection [29, 113, 114], being highly prevalent among adult HIV patients, with a 23% prevalence of NTS found in spleen tissue from adult HIV individuals dying from a different cause [44]. This carriage state postulate is underpinned by the clinical case of a 14 month old child that presented with an invasive infection by *S. typhimurium* after splenectomy due to acute spleen torsion [115]. This is a very close analogy to cases of young children with malaria - an otherwise healthy child, possibly carrier of NTS in the intestine, who suddenly suffers from a hyposplenic state due to a surgical resection of the spleen, which in the case of young African children would be represented by hyposplenism caused by *P. falciparum* malaria infection.

### Malaria infection impairs mucosal inflammatory response

As mentioned, NTS cause high rates of life-threatening bacteraemia in the immune-compromised host and in infants in sub-Saharan Africa, resulting in mortality rates surpassing 20% despite antibiotic treatment [29, 113]. Mucosal barriers represent the first line of defence against bacteria, where lymphoid tissues located between the host and the environment endow with immune cells capable to mount innate and adaptive immune responses against these pathogens [9]. Once bacteria have penetrated the mucosal barrier, the invading micro-organism translocates to the intestinal lymph follicles and the draining mesenteric lymph nodes (MLNs), and some pass on to the reticulo-endothelial cells of the liver and spleen [113]. It has been postulated that malaria infection favours bacteria translocation from the intestinal lumen to the blood, with animal models clearly proving this hypothesis by isolating *S. typhimurium* from liver and spleen by approximately 1,000-fold in animals co-infected with malaria when compared with control animals [114]. These results have been corroborated by the same research group [29] in elegant studies in non-human primate and murine animal models. In these experiments, malaria infection led to increased recovery of *S. typhimurium* from the draining MLNs in mice. Furthermore, malaria infection caused a global suppression of gut inflammatory response and reduced significantly the neutrophil influx to intestinal mucosa, characteristic of NTS infection. IL-10 produced during malaria infection, an anti-inflammatory cytokine whose function is to control the pathogenic inflammatory response against malaria parasites [28], was found to contribute to the suppression of mucosal inflammatory response to invasive NTS in these animal models. However, further mechanisms for bacterial translocation from mucosal surfaces in malaria patients need to be explored; for instance, to study the impact of a reduced amount of circulating MZ B cells in children with malaria in mucosa immune response, since in humans MZ B cells are also located in the subepithelial area of mucosa-associated lymphoid tissues (MALT), including Peyer’s patches and the epithelium of tonsilar crypts [13].

### Vitamin A deficiency

Immune cells have homing molecules resembling ‘immune post-codes’ allowing them to be recruited to specific organs, such as the intestine or skin [116]. Hence, vitamin A plays an important role in the intestinal immune system as a source of retinoic acid (RA), a gut-homing factor for IgA secreting cells in intestinal mucosa [117, 118]. Furthermore, intestinal epithelial cells send signals through RA to DCs in the gut lamina propria promoting IgA class switching in local B cells [118]. It has been shown some decades ago that malnourished vitamin A-deficient rats exhibit impaired migration of recently activated mesenteric lymphocytes, in addition to a marked decrease in the number of IgA-antibody secreting cells and CD4^+^ T cells in the ileum [117]. Moreover, gestational vitamin A deficiency in animal models has been shown to reduce the intestinal immune response by decreasing the number of immune cells in rat offspring [119] and controlling the size of secondary lymph organ and the efficiency of immune responses in the adult mouse offspring [120]. As severe malnutrition is one of the risk factors to develop malaria-IBI co-infection, it is important to evaluate the impact of vitamin A administration in the clinical management of children with malaria and IBI-co-infection in Africa, either as a therapeutic and/or prophylactic measure, especially due to recent evidence of the positive impact of vitamin A and zinc supplementation on malaria morbidity in a study conducted in Ghana [121, 122]. However, in man, the role of vitamin A in malaria pathogenesis is multifaceted, and *P. falciparum* clearly benefits from normal to high vitamin A hepatic stores, possibly depending on scavenged vitamin A metabolites to destabilize host RBC membranes facilitating invasion [123].

### Some loose ends

If splenomegaly and the excessive B cell stimulation in the spleen are the cause of immunological hyposplenism in young children with malaria, it should be expected that patients with other parasitic diseases that cause splenomegaly also develop an increased susceptibility to IBI. There are few reported cases in the literature of increase susceptibility to IBI by NTS in cases of leishmaniasis [124, 125] and schistosomiasis [126, 127]. Although, in the latter co-infection, some more complex mechanisms related to adhesion of bacteria to the *Schistosoma* parasites might be related to recurrent NTS infection. It is interesting that in the case of schistosomiasis, the increased risk to IBI by NTS is also apparently restricted to young children [128]. However, the case of *Plasmodium vivax* malaria and the weak correlation of this infection with IBI is a matter of reflection, especially taking into account that *P. vivax* malaria presents more frequently with massive splenomegaly than *P. falciparum* malaria. That notwithstanding, the concomitant infection of *Salmonella typhi* and *P. vivax* is well established [129–134]. However, the postulated mechanism of this association is the activation of vivax malaria hypnozoites by systemic parasitic and bacterial infections, with little current evidence of *P. vivax* malaria association with other bacterial infections [135]. What could be the explanation for such a prominent splenomegaly without evident immune hyposplenism? A possible mechanism is that in the case of *P. vivax* malaria, the inflammatory response of the B cell subset in the spleen is different, meaning that despite the important capture of infected reticulocytes in the spleen of *P. vivax* malaria patients, the immune reaction is somehow not as strong as in the case of *P. falciparum* malaria (that infects both reticulocytes and mature RBC), therefore not causing a major impact on MZ function. A possible hypothesis to explain this phenomenon could be that *P. vivax* malaria, by infecting reticulocytes, is protected by their CD47 membrane protein, a potent “do not eat me” signal to macrophages in the spleen [136]. Interestingly, CD47 is expressed to a higher level in reticulocytes than in mature RBC [137, 138]. The “do not eat me” signal provided by CD47 has been proven to be strong enough to prevent phagocytosis of antibody opsonized tumour cells when these cells express high quantities of CD47 [139]. Therefore, the expression of CD47 may just be high enough to prevent phagocytosis after *P. vivax* infection. In addition, CD47 has been shown to undergo a conformational change after oxidation, a state that will lead to an “eat me” configuration, which will result in phagocytosis by RP macrophages [136]. It is well known that *Plasmodium* infection leads to reactive oxygen species (ROS) formation, which may lead to oxidation of CD47 and thus the “eat me” configuration of this protein. Reticulocytes, however, have a far better capacity to handle oxidative damage than mature RBC. Thus, infection of reticulocytes by *P. vivax* may not lead to CD47 oxidation and the switch from “do not eat me” to “eat me” signal would not take place, whereas during infection of mature RBC CD47 may be well oxidized. Together, the higher expression of CD47 and the better protection of reticulocytes against ROS generation might explain the accumulation of a big amount of infected reticulocytes in the spleen and the consequent splenomegaly in *P. vivax* malaria, due to the inability of RP macrophages to phagocytose them. In the case of this hypothetical incapacity of the RP to phagocytose *P. vivax*–infected reticulocytes, it is possible that different immunological pathways are triggered when compared to *P. falciparum* malaria, perhaps affecting in a different way or to a lesser extent the MZ immune cell compartment of the spleen.

Additionally, it is important to clarify that the human spleen has remarkable differences compared to the murine spleen, for instance regarding its microcirculation (open in mice, closed and open in humans) and anatomic characteristics (absence of perifollicular zone in mice; absence of marginal sinus in humans) [10, 13, 94]. Also, MZ B cells do not circulate in mice, while they do in humans. Therefore, animal models were included here as a source of valuable information about the dynamic changes of immune cells in this organ in the context of bacterial and parasitic infections, because such studies are not possible to perform in humans. However, this information should be carefully examined and not directly translated to the human case.

## Concluding remarks

There is ample evidence in the scientific literature to support the hypothesis postulated here that African children younger than three years of age might present with immunological spleen dysfunction triggered by *P. falciparum* malaria infection. However, malaria-dependent hyposplenism could possibly affect older children in sub-Saharan Africa as well. This hypothesis is based upon several observations in human spleen histology studies and immune cells in peripheral blood, indicating alterations in the structure and function of the spleen, particularly of the MZ. Animal models, with their acknowledged limitations, were analysed as well in detail and corroborated these findings, opening new questions that need to be urgently addressed in the human host. Testing the dysfunctional spleen hypothesis postulated here would have important implications for bedside care of African children with malaria.

For instance, the development of simple and affordable methods to assess the immune spleen function in children with malaria and other parasitic diseases that colonize the spleen should be a priority because current tools are largely oriented to evaluate the haematological but not the immunological function of this organ. Importantly, as young children lack protective antibodies against several encapsulated bacteria when they face a *P. falciparum* infection, it is necessary to evaluate the role that adjuvant intravenous g-globulin and/or tuftsin treatment could play in helping them overcome their splenic opsonization failure when encountering malaria-IBI co-infections. In this regard, it should be a main concern to accelerate the introduction of several vaccines missing in the expanded programme on immunization in most African countries. In addition, the risk of malaria-IBI co-infection in children from these countries should be taken into account by international organizations and funding sources supporting vaccine introduction in poor countries to orientate their immunization strategies, as well as to increase their investment in research and development of vaccines against NTS.

Assuming that bacteria colonize mucosal surfaces in children and, in the presence of malaria infection, translocate to the bloodstream, it would be important to evaluate the role of probiotics in improving the immune response to IBI in very young children at the first line of defence against these pathogens: the mucosal barrier. Some studies have shown that oral probiotic treatment importantly reduced late onset sepsis in newborns [113], remarkably even against *S. pneumoniae* infection [140]. Of note, the microbiota plays a key role in regulating the host immune system [113, 141], and in the specific case of the genus *Salmonella*, optimizes its response against this micro-organism and mediates pathogen clearance [113]. On the other hand, in some cases prophylactic antibiotic therapy is indicated in children in resource-limited African countries to prevent bacterial infections due to the limited access to health facilities and the difficult medical follow-up. However, as antibiotic treatment, reduces the gut microbiota, it is important to be cautious when given this prophylaxis in children with severe malaria, since the impact on the immune system of the gut could be deleterious, besides the risk of developing antibiotic resistance. Therefore, it is necessary to evaluate the role of probiotics as prophylactics against IBI, as well as adjuvant therapy during and after treatment of bacterial infections, to help the maturity of the immune system and to overcome the effect of antimicrobials in the gut microbiota, respectively, to reduce the morbidity and mortality of bacterial infections in young children.

The time gap between generation of knowledge and its application where is greatly needed is unfortunately long. The aim of this review is to reduce this time gap as much as possible by sharing information considered of value for the malaria research community. The proposed dysfunctional spleen hypothesis needs to be confirmed or rejected by studies in human subjects directly addressing the immune spleen function, since no studies of this kind have ever been performed in African children with malaria. Meanwhile, a general awareness to the health care providers that young children with *P. falciparum* malaria should be treated as hyposplenic patients is advised.

## Abbreviations

^51^Cr: Chromium-51
^99m^Tc: Tecnecio-99 m
BCR: B cell receptor
BM: Bone marrow
CM: Cerebral malaria
CR1: Complement receptor 1
CR2: Complement receptor 2
CRP: Complement regulatory proteins
CXCR5: Chemokine receptor 5
DCs: Dendritic cells
FSV: Functional splenic volume
HIV: Human immunodeficiency virus
HJB: Howell Jolly bodies
IBI: Invasive bacterial infections
IES: Interendothelial slits
Ig: Immunoglobulin
IgA: Immunoglobulin A
IgD: Immunoglobulin D
IgG2c: Immunoglobulin G2c
IgM: Immunoglobulin M
IL-10: Interleukin 10
ILC3: Innate lymphoid cells type 3
MALT: Mucosal associated
MHC: Major histocompatibility complex molecule
MLNs: Mesenteric lymph nodes
MØ: Macroghages
MZ: Marginal zone
MZ B cells: Marginal zone B cells
N_BH_: B cell-helper neutrophils
NK cells: Natural killer cells
NKT: Natural killer T cells
NTS: Non-typhi salmonella
OPSI: Overwhelming post-splenectomy infection syndrome
PALS: Periarteriolar lymphoid sheath
PC: Plasma cells
PMN: Polymorphonuclear neutrophils
PV: Portal vein
PFz: Perifollicular zone
RA: Retinoic acid
RBC: Red blood cell
iRBC: Infected red blood cells
uRBC: Uninfected red blood cells
ROS: Reactive oxygen species
RP: Red pulp
S1P1: Sphingosine 1-phosphate receptor
SCD: Sickle cell disease
SC: SCD heterozygous phenotype
SS: SCD homozygous genotype
SMA: Severe malarial anaemia
SMV: Superior mesenteric vein
SV: Splenic vein
TACI: Transmembrane activator and CALM interactor
TI: T cell independent
TI-1: TI response against polysaccharide microorganism antigens
TI-2: TI response against lipopolysaccharide microorganism antigens
TLR: Toll-like receptors
TNF: Tumour necrosis factor alfa
Transitional T2 B cells: Transitional B cells type 2
VS: Venous sinuses
WP: White pulp.
